# Clinical relevance of cell-free DNA quantification and qualification during the first month after lung transplantation

**DOI:** 10.3389/fimmu.2023.1183949

**Published:** 2023-04-27

**Authors:** Pascal Pedini, Benjamin Coiffard, Nicem Cherouat, Sylvia Casas, Frédéric Fina, Audrey Boutonnet, Jean Baptiste Baudey, Printil Aho, Agnes Basire, Sophie Simon, Coralie Frassati, Jacques Chiaroni, Martine Reynaud-Gaubert, Christophe Picard

**Affiliations:** ^1^ Immunogenetics Laboratory, Etablissement Français du Sang, Marseille, France; ^2^ ADES UMR, Aix Marseille Univ, Marseille, France; ^3^ Aix-Marseille University, Lung Transplant Department, APHM, Marseille, France; ^4^ Medical Direction, CareDx, Brisbane, CA, United States; ^5^ Technical Laboratory, ADELIS Tech, Labege, France

**Keywords:** lung transplantation (LTx), graft rejection (MeSH), infections, chimerism, cell-free nucleic acids (cfNAs)

## Abstract

**Background:**

Many studies have reported the relevance of donor-derived cfDNA (dd-cfDNA) after lung transplantation (LTx) to diagnose and monitor acute rejection (AR) or chronic rejection or infection (INF). However, the analysis of cfDNA fragment size has not been studied. The aim of this study was to determine the clinical relevance of dd-cfDNA and cfDNA size profiles in events (AR and INF) during the first month after LTx.

**Methods:**

This prospective, single-center study includes 62 LTx recipients at the Marseille Nord Hospital, France. Total cfDNA quantification was performed by fluorimetry and digital PCR, dd-cfDNA by NGS (AlloSeq cfDNA-CareDX^®^), and the size profile by BIABooster (Adelis^®^). A bronchoalveolar lavage and transbronchial biopsies at D30 established the following groups: not-injured and injured graft (AR, INF, or AR+INF).

**Results:**

Quantification of total cfDNA was not correlated with the patient’s status at D30. The percentage of dd-cfDNA was significantly higher for injured graft patients at D30 (p=0.0004). A threshold of 1.72% of dd-cfDNA correctly classified the not-injured graft patients (negative predictive value of 91.4%). Among recipients with dd-cfDNA >1.72%, the quantification of small sizes (80-120bp) >3.70% identified the INF with high performance (specificity and positive predictive value of 100%).

**Conclusion:**

With the aim of considering cfDNA as a polyvalent non-invasive biomarker in transplantation, an algorithm combining the quantification of dd-cfDNA and small sizes of DNA may significantly classify the different types of allograft injuries.

## Introduction

In 1948, Mandel and Métais first reported the presence of extracellular nucleic acids in the blood (1). However, due to the lack of sensitive, specific, robust, and reproducible analytical techniques, it was only in 1965 that the first studies identified circulating DNA, cell-free DNA, and extracellular DNA as potential markers of interest in medicine. Almost sixty years later, circulating tumor cfDNA (ctDNA) is at the center of the liquid biopsy concept used routinely in clinical oncology, and fetal-derived cfDNA (cffDNA) has allowed the development of noninvasive prenatal diagnosis (2). In addition to the quantification of cfDNA, the characteristics of the size profiles of cfDNA from fetuses in maternal plasma and the tumor-derived cfDNA molecules (ctDNA) in patient plasma are markers of interest. Indeed, in healthy human subjects, the standard cfDNA fragmentation pattern has a predominant peak at approximately 166 bp and multiples thereof, corresponding to a typical DNA cleavage pattern during apoptosis (3). Fetal DNA and ctDNA have been demonstrated to be shorter (4). The generation of a shorter size would be associated with DNA nuclease activity (5).

In 2019, Knight et al. reported 47 studies (retrospective or prospective) on the analysis of dd-cfDNA after organ transplants (kidney, liver, heart, lung, pancreas) using plasma or urine samples (6). The analyses performed involve the quantification of total cfDNA and an estimation of the percentage of dd-cfDNA by different techniques. Most studies have reported a significant correlation between dd-cfDNA levels and biopsy-proven rejection in kidney, liver, heart, and lung transplants.

In the context of lung transplantation (LTx), De Vlaminck et al. established in 2015 the two basics of the potential utility of dd-cfDNA (7). First, the survival rate after LTx is one of the lowest for all organ transplants, and second, the current diagnostic tests do not distinguish between infection and rejection, which are the two main posttransplant clinical complications. The authors observed a significant relationship between the level of dd-cfDNA and the events of rejection and CMV infection.

Later, Zou et al. (8) performed a digital PCR method according to the HLA mismatch between the donor and the recipient and observed, on the one hand, that there was a significant relationship between dd-cfDNA and acute rejection, but on the other hand, that there was no statistical relationship with bronchiolitis obliterans syndrome (BOS). Other teams have found a correlation between dd-cfDNA and acute rejection (9–12). For other organ transplantation, Agbor-Enoh et al. (13, 14) observed that this relationship is stronger for antibody-mediated rejection (AMR) than for acute cellular rejection (ACR). Moreover, dd-cfDNA was increased before the change in spirometry in recipients for whom the diagnosis of acute rejection will be made, making dd-cfDNA a predictive biomarker of acute rejection. Finally, the authors show that elevated levels of dd-cfDNA before a clinical diagnosis of AMR were associated with a simultaneous increase in DSA levels. Concerning chronic rejection, some authors showed a relationship between dd-cfDNA and the development of chronic lung allograft rejection (CLAD) (15). Finally, in the review by Knight et al. (6), the authors proposed a threshold for the diagnosis of acute rejection at 1%. However, the lack of coherence and consensus among the different preanalytical protocols and the timing for cfDNA analysis is one of the main obstacles to comparing studies and the translation of cfDNA analysis to clinical practice.

This monocentric prospective study investigated the relevance of quantitative and qualitative cfDNA for the diagnosis of early events in LTx in a controlled and reproducible preanalytical process.

## Materials and methods

### Study design, setting, and participants

Sixty-two recipients were included in the Lung Transplant Department of the Aix-Marseille University Hospital, France, between August 28, 2019, and February 10, 2022 (ancillary study from the LARA protocol: NCT03587493) after signing the consent form. The inclusion criteria were age over 18 years old, registered on the national waiting list for a first lung transplant in the Marseille center, regardless of the indication, and benefit from a mono- or bi-lung transplantation. The exclusion criteria were minors, persons protected by law and deprived of their liberty, patients who are not eligible for a social security number, a previous lung transplant, or a patient who has already benefited from another type of transplantation. Finally, patients were excluded from the evaluation if they died or if they did not benefit from a transbronchial biopsy within 60 days after the LTx. The study was approved by an Institutional Review Board (CPP 2018.04.04 ter).

In the study period, all recipients received a standardized immunosuppressive regimen in accordance with our institutional protocol. Induction therapy consisted of intravenous administration of 20 mg of basiliximab on the day of transplant and day 4 post-transplant associated with high-dose methylprednisolone (7.5 mg/kg before implantation). Standard triple maintenance immunosuppressive regimen consisted of intravenous cyclosporine administered immediately after LTx (to obtain a steady-state serum concentration between 300 and 400 ng/ml) and then switched by oral tacrolimus as soon as possible (to maintain trough blood levels between 12 and 15 ng/ml during the first 3 months and around 10–12 ng/ml thereafter), mycophenolate mofetil, and steroids (prednisone) tapered to 0.5 mg/kg/day over the first month of the study period.

Postoperatively, transplant recipients received a prophylactic antibiotic treatment according to their preoperative and/or concomitant infectious status for at least 7 days. Seropositive CMV recipients and higher-risk CMV-mismatched recipients (donor positive and recipient negative) received prophylactic IV ganciclovir or oral valganciclovir as soon as possible, for the entire study period.

### Biological samples

Whole blood samples were collected on the day of transplantation (D0, before surgery) and 15 and 30 days (D15, D30) after transplantation in 8.5 mL Cell-Free DNA Collection tubes (Roche^®^). DNA isolation from the plasma was completed within 7 days. The samples were double-centrifuged (1600 g, 10 min, and 4500 g, 10 min, room temperature). Plasma was stored at -20°C for less than one month before extraction and at -80°C for longer periods before extraction.

### DNA isolation methods

The cfDNA was isolated using a magnetic extraction method (KingFisherTM Flex) with an IDXtract™ Mag kit (ID-Solutions^®^, Grabels France) according to the supplier’s recommendations (16). All cfDNA was stored at 5°C ± 3°C if the PCR was performed immediately or at -20°C for a longer period of storage.

### Fluorimetric cfDNA quantification method

All cfDNA was quantified twice by a QUBIT dsDNA HS Assay kit (Thermo Fisher Scientific^®^, Aalst, Belgium) according to the manufacturer’s recommendations.

### ddPCR cfDNA quantification methods

The commercial ID kit Quant™ cfDNA (ID. Solutions^®^, Grabels, France) and the homemade quantification by ddPCR with RPP30 gene amplification were performed. Quantification of cfDNA was performed by ddPCR using the Bio-Rad QX200 System following manufacturer’s instructions. Absolute quantities of the cfDNA copies were determined using the QX200™ Droplet Reader.

### cfDNA qualification and quantification by BIABooster

Fragment analysis was performed using BIABooster technology (17) (Adelis^®^, Labege, France). The technology was operated automatically on a commercial capillary electrophoresis instrument using electrohydrodynamic actuation. All the samples were treated with 0.1 U/μl RNase before analysis. BIABooster technology enabled the analysis of cfDNA fragments between 75 and 1649 bp, following the manufacturer’s protocol. A reference ladder determines the sizes at each pass. Four peaks and eight areas (<75 bp, 75-111 bp, 111-240 bp, 240-370 bp, 370-580 bp, 580-1650 bp, >1650 bp) were identified and an additional analysis was performed targeting sizes between 80 and 120 bp. The cfDNA concentration (pg/µl) was measured under each area.

### dd-cfDNA determination by NGS AlloSeq cfDNA^®^

The AlloSeq cfDNA kit (CareDx Pty Ltd, WA, Australia) enables relative quantification of the donor-derived cell-free DNA (dd-cfDNA) in a cfDNA sample derived from a transplant recipient. Following cfDNA extraction from plasma, cfDNA was amplified using multiplex PCR that includes PCR primers for 202 single nucleotide polymorphisms. The different reactions, including amplification of targeted regions of interest, indexation, pooling and purification steps were performed, according to the supplier’s recommendations. The sequencing reaction used the MiSeq v3 reagent kit for 150 cycles. Data were analyzed using CareDx AlloSeq cfDNA software, which automatically calculates the dd-cfDNA relative quantification. In each run, a positive control (a previous sample with a known dd-cfDNA value) and a negative control (water) were tested.

### Identification of HLA antibodies against the donor (DSA)

The identification of antibody specificity was carried out using a LABScan 200 Flow analyzer (Luminex Corporation, Austin, TX). The reagents used were LABScreen Single Antigen HLA Class I and Class II (One Lambda, Canoga Park, CA). The tests were carried out according to the manufacturers’ instructions, and the analysis was performed with HLA Fusion 4.4.0 software. The confirmation of DSA was performed by comparing the specificities of the anti-HLA antibodies with the typing of the donor performed using the FluoGene^®^ SSP-PCR technique (Inno-train, Kronberg, Germany) and confirmed by NGS technology (NGmix^®^, EFS).

### Clinical covariables and outcome measures

Clinical data were recorded throughout the study. At inclusion, sex, age, weight, height, blood group, HLA of the recipient and donor, presence of anti-HLA antibodies, underlying lung disease, comorbidities, date of transplantation, type of transplant (single or double), CMV status of the recipient and donor, and type of immunosuppressive induction. At the Day 15 and 30 post-transplant visits, the information collected was the CRP value, the presence of DSA, and the outcome measures: the occurrence of an infection, the occurrence of a CMV infection/disease, and the occurrence of a biopsy-proven rejection. Bronchoalveolar lavage (BAL), bronchial biopsies, and transbronchial biopsies were systematically performed on day 30 and before when infection and/or acute rejection clinically suspected.

Infection was defined by the combination of clinical symptoms, radiological abnormalities (for pulmonary infection), and the identification of a microbe by culture or PCR. CMV infection was defined by the presence of CMV replication in tissue, blood, or other bodily fluids by PCR regardless of symptomatology and CMV disease by the presence of CMV infection that is accompanied by clinical signs and symptoms according to recent guidelines (18). ACR and AMR were histologically defined according to internationally-accepted definitions but not including clinical parameters (19, 20). For ACR, perivascular and interstitial mononuclear infiltrates were graded as A1-4, small airways inflammation/lymphocytic bronchiolitis as B1R or B2R, and large airways inflammation/lymphocytic bronchitis as E1 or E2 (21).

A status was assigned to each patient at D15 and D30, “AR” (acute rejection including ACR, AMR or both labeled as mixed), “INF” (infection), “AR + INF” for “INJURED”, or “NOT-INJURED” (neither rejection nor infection). All outcomes were adjudicated by transplant physicians blinded to dd-cfDNA measurements.

### Statistical analysis

For the comparison of methods, linear regression and correlation tests allowed us to establish the correlation between techniques, and the Bland-Altman test allowed us to compare their concordance. Then, a characterization of the data between them allowed an orientation of the statistical tests performed. For the comparison of two quantitative variables, linear regressions and correlation tests were systematically performed. For two qualitative variables, the chi-square test was performed. To establish the link between quantitative data and qualitative data, the Mann-Whitney test was performed. All tests were performed with XLSTAT Life Sciences software, and the significance level was set at alpha = 0.05. All *Pvalue <*0.05 were considered statistically significant.

## Results

### Patient characteristics

**Table 1** presents a summary of the patient characteristics at inclusion and at D15 and D30 according to their status (not-injured, injured, acute rejection or infection). Sixty-two patients were included in the study, the majority of whom received a bilateral LTx (n=54/62, 87%) for an indication of emphysema (n=24/62, 39%), lung fibrosis (n=19/62, 31%) or cystic fibrosis (6/62, 10%). Sixteen percent of patients had pre-transplant DSAs with an average MFI of 11 300 directed against HLA class I or class II.

**Table 1:** patient’s characteristics at inclusion and at D15 and D30 according to their status.

Recipient demographics	Inclusion	D15 (n=62)					D30 (n=58)**						
		Not-injured	Injured				Not-injured	Injured					
			AR	INF	AR + INF			AR				INF	AR + INF
	n= 62	n=28	n=5	n=25	n=4		n=39	n=6				n=9	n=4
Age (years)													
*Mean (SD)* *Min - max*	53.6 (11) 20-67	57.2 (8) 35-67	55.0 (6) 47-61	49.1 (14) 20-67	54.8 (11) 39-61		53.0 (13) 20-67	52.0 (11) 37-67				57.7 (9) 36-65	54.5 (4) 51-59
Sex: n (%)													
Male	29 (47)	11 (40)	2 (40)	15 (60)	2 (50)		18 (46)	3 (50)				4 (44)	1 (25)
Female	33 (53)	17 (60)	3 (60)	10 (40)	2 (50)		21 (54)	3 (50)				5 (56)	3 (75)
Weight (kg)													
*Mean (SD)* *Min-max*	64.8 (14) 35-100	65.6 (11) 48-88	78.6 (5) 74-86	62.2 (15) 35-100	58.8 (16) 40-78		63.1 (14) 35-100	70.0 (13) 54-88				65.8 (12) 48-86	71.5 (9) 62-81
Height (cm)													
*Mean (SD)* *Min-max*	168 (9.0) 147-185	169 (7) 156-180	168 (10) 157-180	167 (11) 147-185	162 (11) 155-178		168 (9) 147-185	171 (9) 157-180				166 (9) 155-180	165 (10) 154-175
BMI													
*Mean (SD)* *Min-max*	22.9 (3.9) 13.7-32.3	23.0 (3.1) 18.0-29.8	27.8 (1.7) 26.4-30.0	22.1 (4.5) 13.7-32.3	22.0 (4.0) 16.4-24.6		22.3 (4.2) 13.7-32.3	24.1 (4.4) 18.1-30.0				23.6 (2) 19.7-26.5	26.3 (2) 24.2-27.8
ABOD group: n (%)													
O	26 (42)	10 (36)	3 (60)	11 (44)	2 (50)		16 (41)	3 (50)				3 (33)	2 (50)
A	25 (40)	13 (46)	1 (20)	11 (44)	0 (0)		15 (38)	1 (17)				5 (56)	2 (50)
B	8 (13)	4 (14)	0 (0)	2 (8)	2 (50)		5 (13)	2 (33)				1 (11)	0 (0)
AB	3 (5)	1 (4)	1 (20)	1 (4)	0 (0)		3 (8)	0 (0)				0 (0)	0 (0)
D +	58 (94)	25 (89)	5 (100)	24 (96)	4 (100)		36 (92)	6 (100)				8 (89)	4 (100)
D -	4 (6)	3 (11)	0 (0)	1 (4)	0 (0)		3 (8)	0 (0)				1 (11)	0 (0)
Lung transplantation: n (%)													
Double lung transplantation	54 (87)	24 (86)	5 (100)	21 (84)	4 (100)		33 (85)	5 (83)				9 (100)	4 (100)
Simple lung transplantation	8 (13)	4 (14)	0 (0)	4 (16)	0 (0)		6 (15)	1 (17)				0 (0)	0 (0)
Indications: n (%)													
Emphysema - COPD	24 (39)	14 (50)	2 (40)	7 (28)	1 (25)		15 (38)	1 (17)				4 (45)	2 (50)
Lung fibrosis	19 (31)	7 (25)	3 (60)	7 (28)	2 (50)		12 (31)	2 (33)				3 (33)	2 (50)
Cystic fibrosis	6 (10)	1 (4)	0 (0)	4 (16)	1 (25)		4 (10)	1 (17)				1 (11)	0 (0)
Bronchial dilatation	4 (6)	2 (7)	0 (0)	2 (8)	0 (0)		3 (8)	0 (0)				1 (11)	0 (0)
Alpha anti trypsin deficit	1 (1.5)	1 (4)	0 (0)	0 (0)	0 (0)		0 (0)	1 (17)				0 (0)	0 (0)
Sarcoidosis	1 (1.5)	1 (4)	0 (0)	0 (0)	0 (0)		0 (0)	1 (17)				0 (0)	0 (0)
Other	7 (11)	2 (7)	0 (0)	5 (20)	0 (0)		5 (13)	0 (0)				0 (0)	0 (0)
CMV status: n (%)													
D+/R+	27 (44)	15 (54)	3 (60)	8 (32)	1 (25)		16 (41)	3 (50)				4 (45)	1 (25)
D-/R-	22 (35)	8 (29)	1 (20)	12 (48)	1 (25)		13 (33)	2 (33)				3 (33)	3 (75)
D-/R+	11 (18)	5 (17)	1 (20)	3 (12)	2 (50)		8 (21)	1 (17)				2 (22)	0 (0)
D+/R-	2 (3)	0 (0)	0 (0)	2 (8)	0 (0)		2 (5)	0 (0)				0 (0)	0 (0)
CRP (mg/L)													
*Mean (SD)* *Min - max*							15.6 (26) 0.3-126.6	6.12 (7) 0.9-19.5				49.8 (91) 4.3-255.2	92.9 (153) 2.5-269.7
DSA D0: n (%)													
No DSA	52 (84)	24 (86)	3 (60)	24 (96)	1 (25)		35 (90)	5 (83)				6 (67)	2 (50)
Class I	6 (10)	3 (10)	2 (40)	0 (0)	1 (25)		2 (5)	1 (17)				1 (11)	2 (50)
Class II	4 (6)	1 (4)	0 (0)	1 (4)	2 (50)		2 (5)	0 (0)				2 (22)	0 (0)
MFI total *Mean (SD)* *Min-max*	11,300 (20,468) 800-68,000	5,675 (7,044) 800-16,000	4,450 (566) 2700-3500	1,100	27,667 (35,020) 5,000-68,000		4,875 (7,424) 800-16,000	2,700				6,433 (3,108) 4,300-10,000	35,750 (45,608) 3,500-68,000
DSA D15: n (%)				n=57*				n=54***					
		n=28	n=5	n=20	n=4		n=35	n=6				n=9	n=4
No DSA		14 (50)	0 (0)	12 (60)	0 (0)		21 (60)	1 (17)				2 (25)	0 (0)
Class I		3 (11)	2 (40)	3 (15)	2 (50)		4 (11)	1 (17)				0 (0)	4 (100)
Class II		5 (18)	1 (20)	4 (20)	1 (25)		6 (17)	0 (0)				4 (50)	0 (0)
Class I + II		6 (21)	2 (40)	1 (5)	1 (25)		4 (10)	4 (66)				2 (25)	0 (0)
MFI total *Mean (SD)* *Min-max*		9,264 (8,387) 550-23,900	27,600 (11,120) 1,500-27,600)	4,036 (3,314) 700-10,400	13,425 (15,942) 500-36,600		6,025 (6,187) 500-18,700	7,475 (6,564) 3,800-17,300				8,333 (6,839) 700-19,800	10,450 (17,444) 1,100-36,600
AR type: n (%)													
Acute cellular rejection (ACR)			1 (20)		2 (50)			3 (50)					0 (0)
*A score* *B score* *E score*			*A1* *BX* *NR*		*A1* *NR* *NR*	*A1* *BX* *E0*		*A0* *B0* *E1*	*A1* *BX* *NR*		*A0* *B0* *E1*		
Antibody-mediated rejection (AMR)			3 (60)		1 (25)			1 (17)					1 (25)
Mixed rejection (ACR + AMR)			1 (20)		1 (25)			2 (33)					3 (75)
*A score* *B score* *E score*			*A0* *BX* *E1*		*A4* *BX* *E0*			*A2* *BX* *NR*		*A1* *NR* *NR*			

* 4 patients did not have DSA analysis and one patient died before; **2 patients died, 2 patients have a non-informative biopsy; *** 4 patients did not have DSA analysis. AR, acute rejection; INF, infection; SD, standard deviation; COPD, chronic obstructive pulmonary disease; CMV, cytomegalovirus; D/R, donor/recipient; DSA, donor specific.

**Figure 1:**
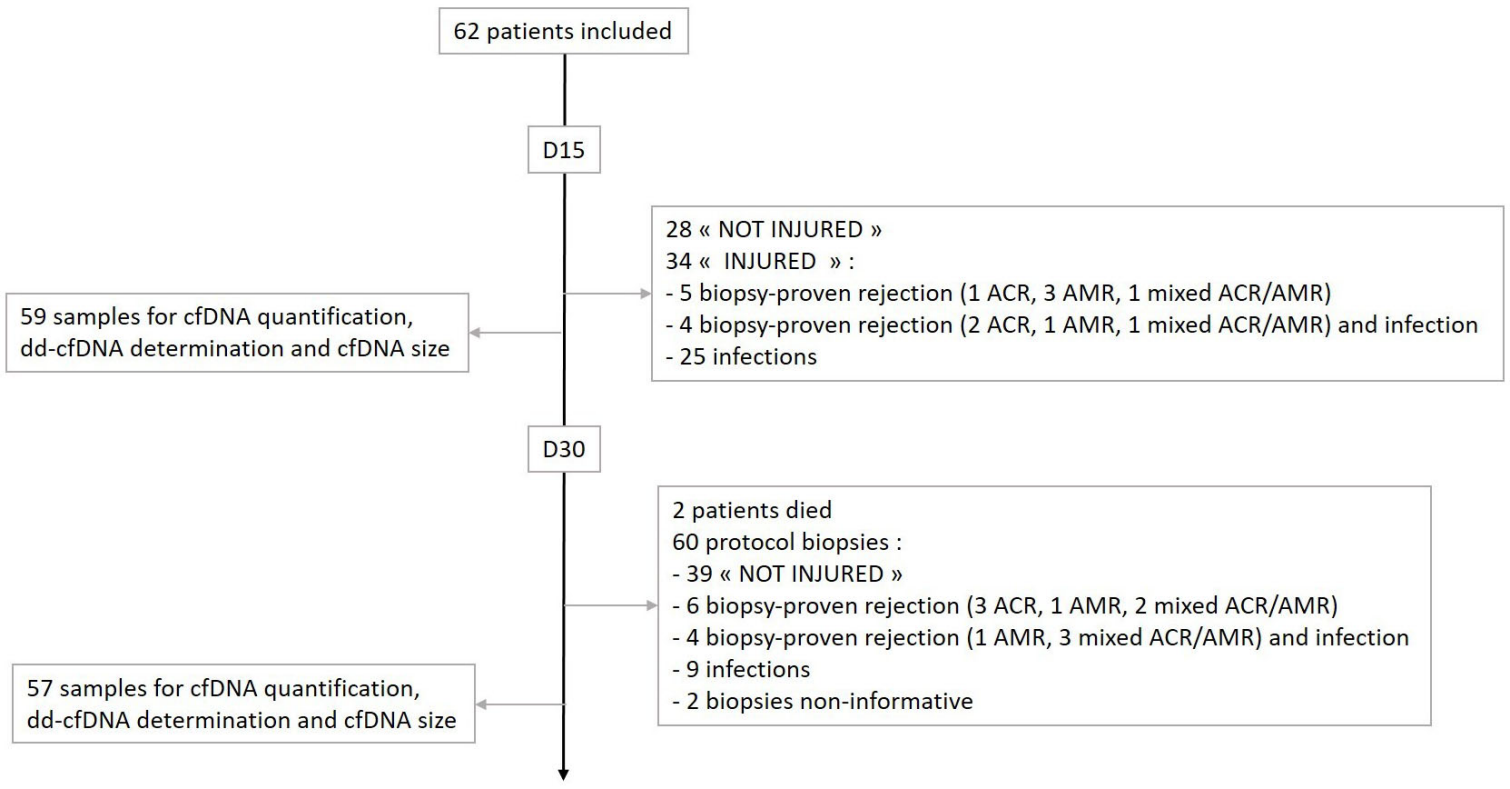
Patient and study sample flowchart.

On day 15, 28/62 (45%) patients had neither acute rejection nor infection, whereas 25/62 (40%), 5/62 (8%), and 4/62 (6%) had infection, acute rejection, or both, respectively. On day 30, 2 patients died, and 2 patients had uninformative biopsies. Forty out of 58 (70%) of the patients were not-injured, while 9/58 (16%), 8/58 (14%), and 1/58 (2%) had acute rejection, infection, or both, respectively (**Figure 1**).

**Table 2** describes the biological and clinical characteristics of AR and INF. Among the AR, five were mixed acute rejection, 3 ACR, and 2 AMR. Only one biopsy showed a histopathological score of 3. Four AR were associated with INF. The INF were either of lung origin, or sepsis (6 cases). The microbiological pathogens were various.

Table 2Description of injured graft patients at D30.

**Table 2A:** Description of injured graft patients at D30: Biological characteristics of Acute Rejection.

Patient	Associated infection	Type of rejection	Biopsy score			Presence of DSA	Total MFI	Type of DSA	%dd-cfDNA	%80-120bp
1	Yes	Mixed	A3	BX	NR	Yes	45,400	A1 A68 B8 B18	3.00%	10.63%
2	Yes	Mixed	A0	B1	E0	No			2.90%	4.36%
3	Yes	AMR	A0	B0	E0	Yes	600	B44	2.70%	4.33%
4	Yes	Mixed	A1	BX	E0	No			3.10%	6.42%
5	No	Mixed	A2	BX	NR	Yes	4,000	B50 Cw5 DR53	5.10%	1.06%
6	No	Mixed	A1	NR	NR	Yes	3,500	A74 DQA1*02	2.20%	1.99%
7	No	ACR	A0	B0	E1	Yes	4,850	A2 DQ8	4.30%	3.41%
8	No	ACR	A1	BX	NR	Yes	7,950	DR15 DQ2 DQ6	0.78%	2.51%
9	No	AMR	A0	B0	E0	Yes	750	B8	4.26%	2.21%
10	No	ACR	A0	BX	E1	No			9.60%	2.04%

ACR, acute cellular rejection; AMR, antibody-mediated rejection; NR, not realized, DSA, donor specific antibodies; MFI, mean fluorescence intensities.

**Table 2B:** Description of injured graft patients at D30: Clinical and biological characteristics of infections.

Patient	Associated rejection	CRP mg/L	Infection diagnosis and microorganism	Treatment	%dd-cfDNA	%80-120bp
1	Yes	269.7	VAP *Enterobacter cloacae*, BSI *Staphylococcus epidermidis*	ertapenem + vancomycin	3.00%	10.63%
2	Yes	2.5	Pneumonia *Klebsiella aerogenes* and *MRSA*	cefepime + linezolid	2.90%	4.36%
3	Yes	6.5	Septic shock without documentation	piperacillin/tazobactam + vancomycin	2.70%	4.33%
4	Yes	NR	Septic shock *Escherichia coli*	cefotaxime	3.10%	6.42%
5	No	NR	Translocation on fecal impaction, *Pseudomonas aeruginosa* and *Stenotrophomonas maltophilia*	amphotericin B + ciprofloxacin + meropenem	2.40%	11.02%
6	No	23.3	Pneumonia *Enterobacter cloacae*	untreated	1.80%	7.22%
7	No	23.1	Presence of mycelial filaments on biopsy	fluconazole	4.50%	4.55%
8	No	4.3	Pneumonia *Pseudomonas aeruginosa* and *Achromobacter xylosoxidans*, BSI *MSSA*	imipenem/cilastatin	3.10%	8.68%
9	No	7.6	Pneumonia *Staphylococcus epidermidis*	piperacillin/tazobactam	2.40%	3.74%
10	No	NR	BSI *Serratia marsescens*	cefepime + ciprofloxacin + gentamicin	1.10%	10.10%
11	No	25.2	BSI *Staphylococcus epidermidis*, VAP *Proteus mirabilis*	cefepime + vancomycin	0.90%	11.91%
12	No	255.2	VAP *Corynebacterium*	piperacillin/tazobactam + linezolid	3.30%	2.44%
13	No	10	BSI *Staphylococcus haemolyticus*	vancomycin	2.70%	2.97%

VAP, Ventilator-associated pneumonia; BSI, Bloodstream infections; MRSA, Methicillin-resistant S. aureus; MSSA, Methicillin-sensitive Staphylococcus aureus; NR, not realized.

Transplantation status (not-injured *vs*. injured) at D15 was correlated with the presence of DSA on the day of transplantation (p=0.003) and at D15 (p=0.007). In “injured group”, there was a stronger level of significance between the AR and non-AR groups (day of transplantation, p=0.002; D15, p=0.003). Transplant status at D30 did not correlate with any other parameter, not even with the presence of pre-transplant DSA (p=0.161), or DSA at D15 (p=0.080) or D30 (p=0.275) (**Table 3**).

**Table 3:** Univariate analysis of factors associated to clinical status, cfDNA quantification, % dd-cfDNA, % 80-120 pb size at D15 and D30.

	D15 status	cfDNA quantification D15	dd-cfDNA (%) D15	80-120 pb (%) D15	D30 status	cfDNA quantification D30	dd-cfDNA (%) D30	80-120 bp (%) D30
Sex								
Age			P					
Weight								
Height								
Blood group								
Underlying lung disease								
Type of transplant			P					
CMV status								
DSA D0	P							
DSA D15	P							
DSA D30								
CRP D30								
Status D15								
Status D30							P	P

Black box: p>0.15; Grey box: trend (0.05<p<0.15); White box: p<0.05, “P” : positive correlation.

### Total cfDNA quantification methods are not associated with early transplant events

**Figure 2:**
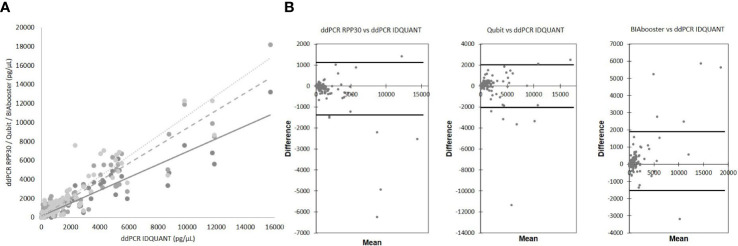
Comparison of total cfDNA quantification techniques. **(A)** correlation between techniques ddPCR IDQUANT and ddPCR RPP30 (solid line, y = 0.6776x + 127.9; R² = 0.8822, p<0.0001), Qubit (dash line; y = 0.9266x + 137.99; R² = 0.8936, p<0.0001) and BIAbooster (dotted line; y = 1.0656x + 39.642; R² = 0.871, p<0.0001) **(B)** Bland-Altman plots; concordances between techniques ddPCR IDQUANT and ddPCR RPP30, Qubit and BIAbooster; 95% CI (black solid line).

A graphical representation revealed a correlation among the four techniques used for quantification (**Figure 2A**). The ddPCR RPP30 and BIAbooster were the most strongly correlated (r²=0.934), while the ddPCR RPP30 and Qubit were the least (r²=0.683). The Pearson correlation test showed a p-value<0.0001 for all techniques.

Analysis with a Bland-Altman plot (**Figure 2B**) showed good agreement between the techniques. Some outliers were present, especially for the high values of quantification, which were not adapted to the principle of rare events of digital PCR.

No statistical correlation was identified between the quantification of total cfDNA and the parameters collected at D15 and D30, particularly with the transplantation status (**Table 3**).

### The level of %dd-cfDNA at D30 is associated with the transplant status

**Figure 3:**
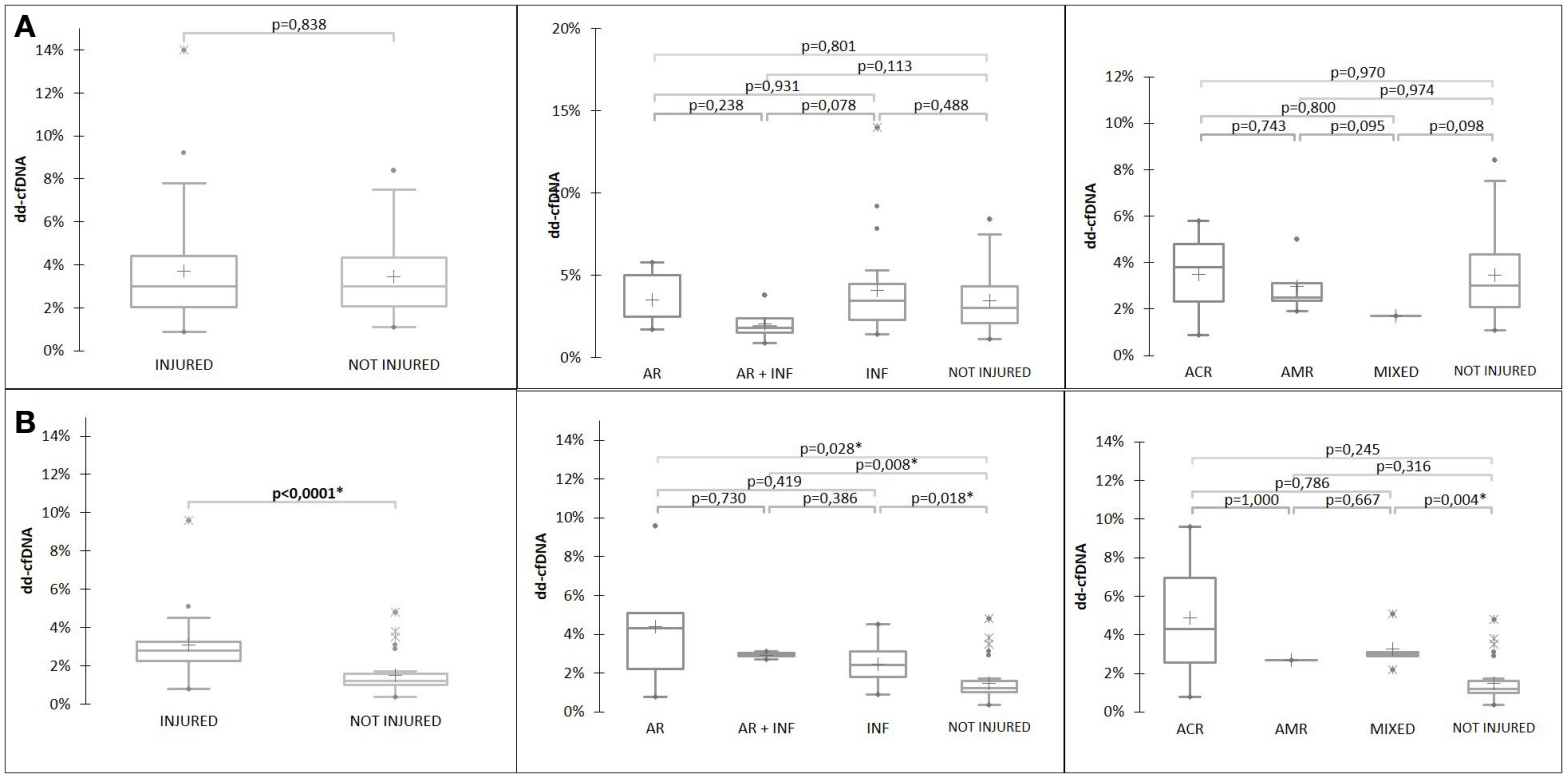
%dd-cfDNA at D15 **(A)** and at D30 **(B)** according to patient status.

In the early post-transplant period, there was a high level of %dd-cfDNA, probably related to the surgery. Indeed, at D15, all patients had %dd-cfDNA levels above 1%, and although there was no significant difference regarding the status of the transplantation (**Figure 3A**), the %dd-cfDNA level was significantly higher (p=0.031) with double transplantation (**Supplementary Figure**).

The level of %dd-cfDNA at D15 was statistically decreased with the age of the patient (p=0.017), (**Table 3**).

**Figure 4:**
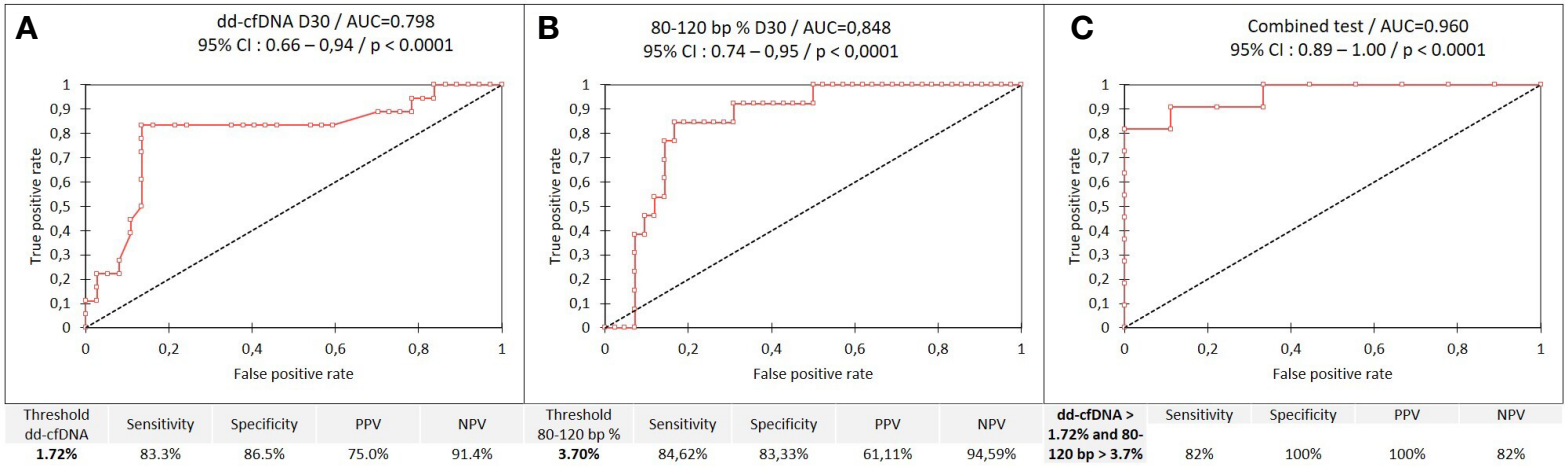
ROC curves; **(A)**, %dd-cfDNA between not-injured and injured graft patients **(B)**, %80-120bp size between not-injured and injured graft patients and **(C)**, combined test between infected and uninfected groups.

The level of %dd-cfDNA at D30 was higher when the patient was injured graft than when the patient was not-injured graft (p<0.0001). Moreover, there is a significant difference (**Figure 3B**) between the AR (p=0.028), INF (p=0.018), and “INF + AR” (p= 0.008) groups compared to the “not-injured”, but no difference between the “AR” and “INF” groups (p=0.419). A ROC analysis (**Figure 4**) showed an area under the curve (AUC) of 0.798 (p<0.0001), establishing a threshold of 1.72% of %dd-cfDNA between not-injured and injured graft patients (**Figure 4A**). Under these conditions, the negative predictive value (NPV) was 91.4% and the positive predictive value (PPV) was 75.0%. Interestingly, the mean %dd-cfDNA levels tended to be higher in AMR than in ACR (mean value: 3.1% *vs*. 1.9%, p=0.10). The *de novo* or pre-transplant DSA, regardless of the level and the type (Class I *vs*. class II HLA antibodies) detected at D15 or D30, was not correlated with the level of %dd-cfDNA at D30 (**Table 3**).

### The proportion of cfDNA with sizes of 80 to 120 bp is associated with infection at D30

**Figure 5:**
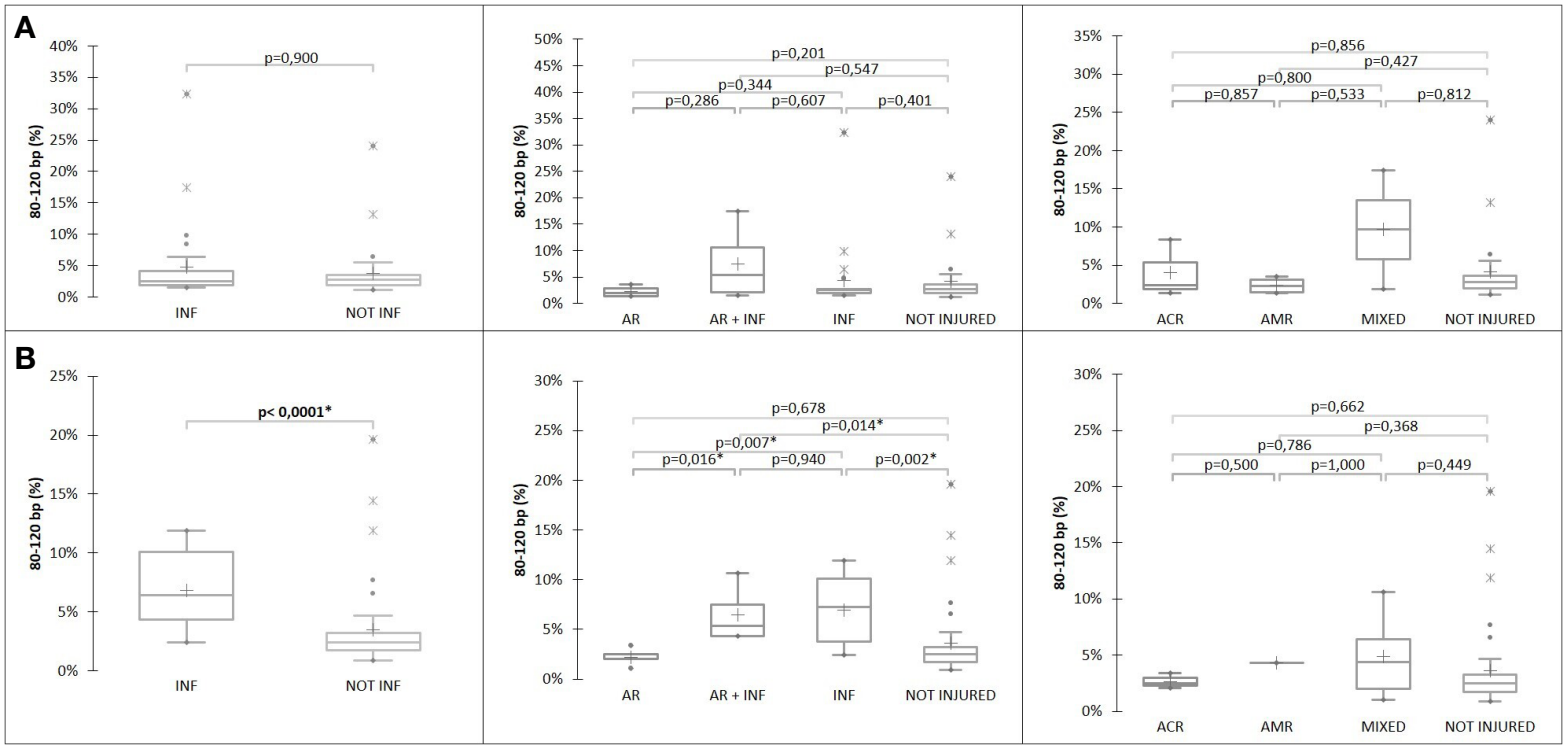
%80-120bp cfDNA at **(A)** D15 and **(B)** D30 according to patient status.

There was no association between the proportion of each DNA fragments size, included 80-120bp and the status of the transplant at D15 (**Figure 5A** and **Table 3**). However, the level of %dd-cfDNA tended to correlate with small cfDNA sizes, 80-120 bp (p=0.010). At D30, the proportion of cfDNAs for sizes 80 to 120 bp correlated with INF, allowing us to differentiate between INF and NO-INF patients (p=0.005, **Figure 5B**). A ROC analysis considering the percentage of 80-120 bp cfDNA sizes associated with the INF group and NO-INF group indicated an AUC of 0.848 (p<0.0001), a NPV of 94.6% and a PPV of 61.1% for a threshold >3.7% (**Figure 4B**). In particular, the test correctly identified 12 of the 14 INF groups (85%), without excluding samples in which AR occurred together with INF.

### Combining the association of %dd-cfDNA and %80-120bp cfDNA size is associated with INF occurrence

**Figure 6:**
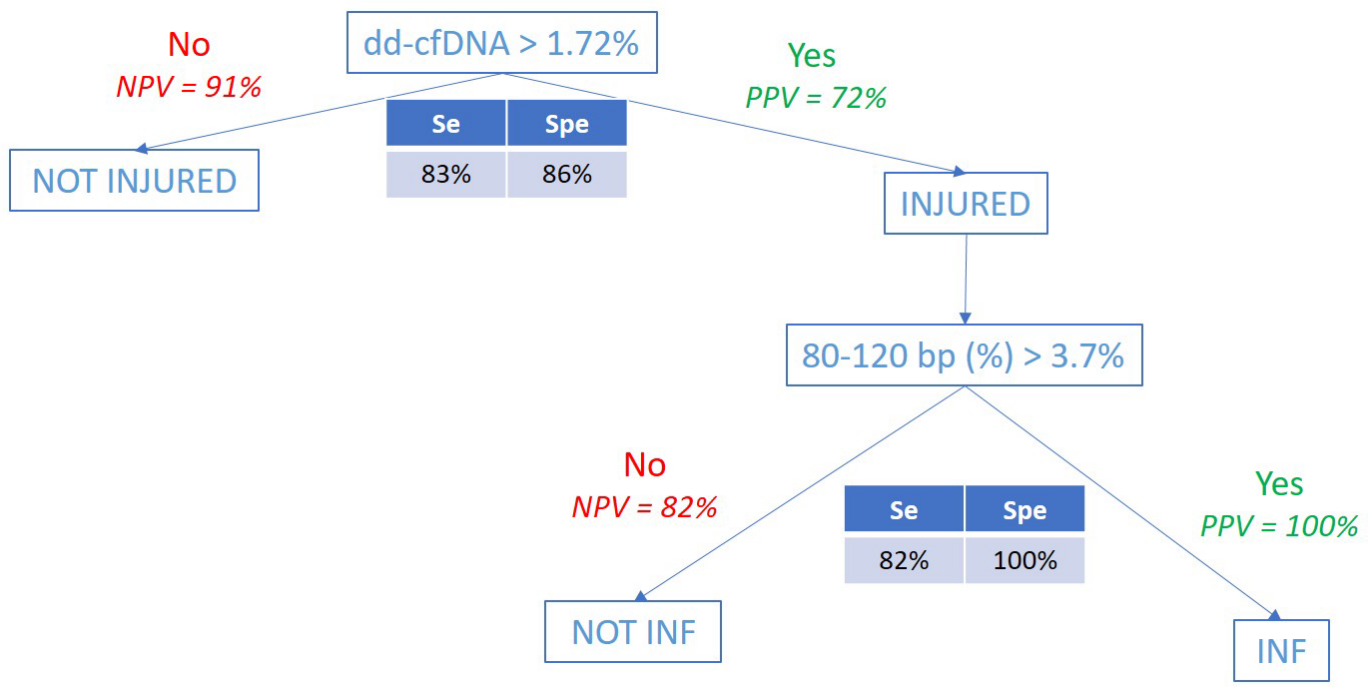
Analytical performance of the combined test (%dd-cfDNA and %80-120bp cfDNA).

At D30, a ROC analysis (**Figure 4C**) considering the percentage of 80-120 bp cfDNA sizes associated with the INF group and NO-INF group when the %dd-cfDNA was >1.72% showed an AUC of 0.960 (p<0.0001). Under these conditions, the positive predictive value (PPV) was 100%, and the NPV was 82%. An analysis combining the two biomarkers dd-cfDNA >1.72% and 80-120 bp cfDNA size >3.7% produced the analytical performance described in **Figure 6**. Interesting, the CRP values tended to correlate only with the %dd-cfDNA and %80-120bp cfDNA sizes (p=0.15 and p=0.08, respectively) (**Table 3**).

## Discussion

This is, to our knowledge, the first study that attempted to identify the events at D30 post-LTx by combining the level of dd-cfDNA and the cfDNA size fragment profile. In contrast, the total quantification of cfDNA, regardless of the quantification method used, did not seem relevant for the diagnosis of an early transplant event. As proof, there was no statistical variation in this quantification between D15 and D30 after transplantation (data not shown), suggesting that the patient was in a physiological state of significant cell death or active cfDNA release. It would be interesting to follow this total quantification longer in the post-LTx follow-up to improve our understanding of the pathophysiology of cfDNA in organ transplantation.

Our study showed that the %dd-cfDNA analysis would be an efficient biomarker for the diagnosis of early events past the first 15 days after transplantation. As a reaction to surgery and ischemia-reperfusion, we observed, like other authors, a very high %dd-cfDNA level in the first days of transplantation since all patients exceeded 1% at D15 (7, 8, 14, 22, 23) and that this level was more elevated when the patient received a double lung transplant (24). Although not related to the medical status of the transplant, the %dd-cfDNA level at day 15 was correlated with the percentage of small fragments 80-120 bp in size, suggesting that the donor-derived cfDNA is smaller than that of the recipient.

However, at D30, the %dd-cfDNA levels were significantly lower, and a threshold of 1.72% of %dd-cfDNA was associated with a significant negative predictive value to differentiate not-injured from injured patients, indicating that a %dd-cfDNA value lower than 1.72% would indicate a not-injured transplant with high confidence. This value is very close to those of other studies. Indeed, a review shows thresholds of %dd-cfDNA around 1%, but the clinical events were detected at more distant post-LTx times, and the pre-analytical and analytical processes are unique to this study. Thus, the determination of %dd-cfDNA by clinical-grade NGS allows reproducibility, automation, and above all, the screening of 202 markers without requiring any donor DNA, allowing for good precision. For example, Zou et al. (8) and Sorbini et al. (25) used ddPCR with unitary markers related to HLA-DR mismatches between the recipient and the donor, or Agbor-Enoh et al. (13) used NGS but with the need for donor DNA. According to a review by Knight et al. (6), all previous studies use technologies for which a donor DNA sample is needed.

In our study, a %dd-cfDNA value higher than 1.72% could not differentiate between AR and INF. The threshold analysis was the same when considering or not considering pauci-symptomatic patients and in A1 rejection. Interestingly, as noted by others (13), the mean %dd-cfDNA levels in AMR were significantly higher than that of ACR, independent of the histologic stage and clinical severity of AR. However, *de novo* or pre-transplant DSA did not induce a change in the %dd-cfDNA levels. In our study, DSA pathogenicity as part of complement activation or the strength of the FcR-mediated ADCC was not investigated. Our teams and others showed that DSA detected after three months was potentially more strongly associated with chronic rejection occurrence in LTx (26). Furthermore the humoral alloimmune-mediated injury seems to be a less important contributor to death-censored graft loss in lung transplant recipients than in kidney transplant recipients (27).

Some cases considered not-injured (without infection and without acute clinical and histological rejection) at D30 had a high percentage of %dd-cfDNA. Several studies suggest a greater sensitivity of %dd-cfDNA for the detection of clinically silent allograft injury compared to bronchoscopy with transbronchial biopsy; these cases surely would require more frequent clinical follow-up (14). However, in our study, none of these cases reported clinical AR within the first 3 months (data not shown). Thus, our study suggests that in practice, a positive %dd-cfDNA level may serve as a trigger for bronchoscopy and other tests, such as radiologic, histopathologic, and BAL data, to identify allograft injury. In contrast, a %dd-cfDNA value lower than 1.72% may limit the need for clinical and biological investigation.

Interestingly, in our study, the last informative marker related to %dd-cfDNA was the proportion of small cfDNA (80-120bp), which correlated with INF at a threshold of 3.7%. Indeed, when the cutoff was >3.7%, the patients were infected in all cases, and when the cutoff was <3.7%, AR was more frequent than INF. The positive microbiology was detected not exclusively by different means, such as bronchial aspiration, BAL, histopathology, and blood culture. All blood cultures were positive within 5 days of collection. Several pathogen species, the most found types, were detected, suggesting an absence of a correlation between the pathogen species and the values of the two cfDNA markers. In the two cases with a fragment size 80-120 bp <3.7%, the infections were known to be present for longer than 5 days with adapted antibiotic treatment. Thus, these data may indicate that %dd-cfDNA, and probably even more so the %80-120bp cfDNA size, is elevated in the setting of relevant local lung infection.

Our hypothesis is that small cfDNA fragment sizes are an indirect marker of graft damage, probably as part of the oncology process, due to the activation of DNAse I in the tissue. However, its specificity for the infected tissue may not be that of the lung graft, as evidenced by the high percentage of size fragments, while the %dd-cfDNA value is below that of the threshold. Additionally, it would be relevant to perform the chimerism test specifically on these small sizes, by comparing the results of chimerism from fragments of small sizes and those of large sizes. This size selection would increase the %dd-cfDNA value and make it possible to be more specific for infectious lung lesions. The study by Bazemore et al. (28) showed that %ddcfDNA is higher when isolating high-risk pathogens known to increase the risk of allograft dysfunction. Our study confirms that the level of %ddcfDNA is higher and that %80-120bp is also increased in the case of infection by a high-risk microorganism.

In contrast to other authors (25), the CRP parameter was not associated with %dd-cfDNA or the %80-120bp cfDNA size, suggesting that these infection markers could be interesting when following a local infection evolution, independent of the classical systemic inflammation biochemical marker parameters. Thus, an algorithm approach combining %dd-cfDNA and the %80-120bp cfDNA size is mainly useful to determine an infection occurrence and potentially the source of the infection.

There are few limitations to this study. First, it is a study with preliminary results based on a small cohort. Then the clinical data collected are limited. In a future study, it would be interesting to study the impact of ischemia time, ECMO and length of mechanical ventilation. Also, the impact of the donor-related parameters will be interesting to consider, such as the age of the donor.

Our study shows that the %dd-cfDNA value, measured at D30, is correlated with early transplant events. This analysis is not able to differentiate between infection and rejection, as some authors do. To discriminate the causes of the recipient organ injury (infection in particular), the size of the cfDNA is very promising. A threshold of 3.7% for small fragment sizes from 80 to 120 bp gave a satisfactory positive predictive value to detect infection. This marker could be useful to research local or systemic infections with high-risk pathogens associated or not associated with downstream allograft dysfunction and to follow this clinical evolution after treatment. However, the %dd-cfDNA was the only specific marker of allograft injury. Chimerism analysis of small fragment sizes could reveal the lung origin of infections.

In conclusion, our study suggests, on the one hand, that cfDNA is a very attractive non-invasive marker for the follow-up of transplanted patients and, on the other hand, that this biomarker could finally participate in the decision strategy to perform lung biopsies at D30. Nevertheless, due to its lack of specificity for rejection, it is more prudent to consider this new diagnostic tool as a polyvalent biomarker (%dd-cfDNA, fragmentomics, epigenetic signatures …). As the review by Jimenez-coll et al. (29), our study is a step in the evolution of the biology of organ transplantation towards personalized and predictive medicine based on the use of different panels of biomarkers both before transplantation and for its monitoring.

This study must be validated with a larger replication cohort and by a multicenter study to test the reproducibility of the analytical protocol and to observe the potential “center” effects of the surgical type, which are particularly sensitive when studying early events.

## Data availability statement

The original contributions presented in the study are included in the article/**Supplementary Material**. Further inquiries can be directed to the corresponding author.

## Ethics statement

The study was approved by an Institutional Review Board (CPP 2018.04.04 ter). Sixty-two recipients were included in the Lung Transplant Department of the AixMarseille University Hospital, France, between August 28, 2019, and February 10, 2022 (ancillary study from the LARA protocol: NCT03587493) after signing the consent form.

## Author contributions

PP supervised the study and performed the technical and statistical analyses. BC and MR-G collected the clinical data from the patients. NC, JB, PA performed the extraction and quantification of cfDNA. SC, FF, AB participated in the technical expertise. ABa, SS, CF, JC, and CP assisted in the interpretation of the results. All authors contributed to the article and approved the submitted version.
