# Supplementary figures and images for: Clinical relevance of cell-free DNA quantification and qualification during the first month after lung transplantation
Source: Front Immunol. 2023 Apr 27;14:1183949. doi: 10.3389/fimmu.2023.1183949 (PMC10174290; doi:10.3389/fimmu.2023.1183949)

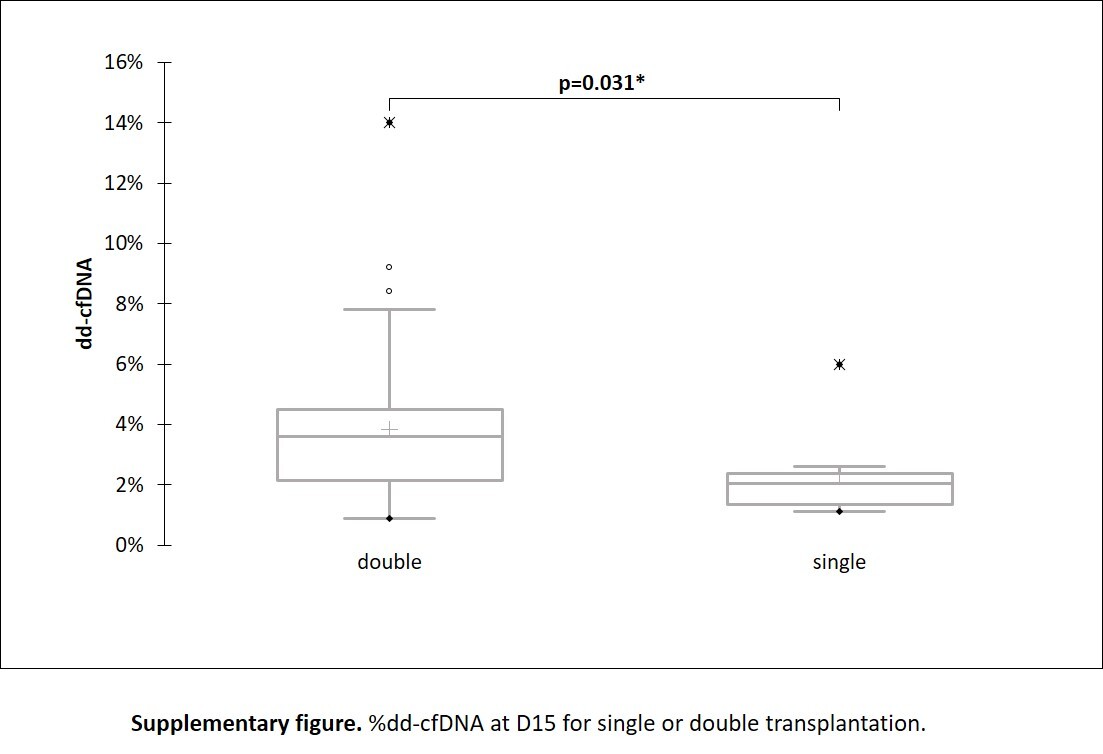

Supplement: Supplementary file 1 [file Image_1.jpeg]
