# Supplementary material for: Clinical relevance of cell-free DNA quantification and qualification during the first month after lung transplantation
Source: Front Immunol. 2023 Apr 27;14:1183949. doi: 10.3389/fimmu.2023.1183949 (PMC10174290; doi:10.3389/fimmu.2023.1183949)
Supplement: Supplementary file 2 [file Table_1.docx]

SUPPLEMENTAL MATERIALS AND METHODS

The study of cfDNA needs to be based on reliable analytical techniques. On the one hand we compared different processes and on the other hand we used a robust and reproducible pre-analytical and analytical protocol by using CEIVD material or controlled by internal controls.

Biological samples

Whole blood samples were collected on the day of transplantation (D0, before surgery) and 15 and 30 days (D15, D30) after transplantation in 8.5 ml cell-free DNA collection tubes (Roche®). The Cell-Free DNA Collection Tube is a direct-draw tube for the collection, stabilization and transportation of whole blood specimens. It effectively prevents blood coagulation, prevents cell lysis and preserves nucleated cells to enable efficient analysis of cell-free DNA (cfDNA). The Cell-Free DNA Collection Tube is CE-IVD formats. DNA isolation from plasma was performed within 7 days. The samples are double centrifuged (1600g, 10min, RT and 4500g, 10min, RT). Plasma is stored at -20°C for less than one month before extraction and at -80°C for longer periods before extraction.

DNA isolation methods

The cfDNA was isolated using a commercial magnetic extraction method (KingFisherTM Flex) with IDXtract^TM^ Mag kit (ID-Solutions®, Grabels France) according to the supplier's recommendations. The plasma volume is 2mL and the elution volume is 120µL. A defined quantity of an exogenous internal control (ICE) is introduced into each extraction to serve as extraction quality control. A negative extraction control (NEC) with PBS and a positive control (TPC) (Manufacturer's Positive Control Indicator) are used to control the absence of contamination and the correct extraction. All cfDNA were stored at 5°C ± 3°C if the PCR is performed immediately or at -20°C for longer storage.

Fluorimetric cfDNA quantification method

All cfDNA were quantified twice by QUBIT dsDNA HS Assay kit (Thermo Fisher Scientific®, Aalst, Belgium), according to the manufacturer’s recommendations. The addition of 190µL of reaction mix to 10µL of cfDNA or standards (high and low) allows the measurement of the cfDNA concentration in ng/µL.

ddPCR cfDNA quantification methods

The commercial kit IDQuant™ cfDNA (ID.Solutions®, Grabels, France) is a standardised detection and quantification kit using ddPCR that allows the amplification of a target sequence of the human genome present at a rate of one copy per haploid genome. It is a relative quantification duplex system which allows, for each sample, the simultaneous amplification of extracted cfDNA and an exogenous internal control (ICE). The ICE control allows to evaluate the target extraction efficiency for each sample and thus to normalize the inter-test fluctuations for the same individual during a follow-up over time. By this standardization, IDQuant™ allows monitoring of the concentration of circulating DNA over time for the same individual. The reaction mix consists of 8 µL cfDNA and 13µL ARM (Reaction Mixture Amplification) containing Taq Polymerase and oligonucleotides for the detection of cfDNA (FAM) and ICE (HEX). Each ddPCR quantification includes the positive extraction control (TPC) to validate the extraction process and the positive quantification control (TPC-IDQuant) which is a synthetic DNA calibrated to a known concentration, to validate the amplification of the target and the ICE control. Moreover, there are the negative extraction control (NEC) to control the absence of extraction contaminant and the negative amplification control (NAC) which is nuclease-free water, to control the absence of aerosol contaminant.

The quantification of the cfDNA was performed using the QX200™ Droplet Digital PCR System (BioRad, Hercules, CA, USA) following the manufacturer's instructions. The ddPCR reaction mixture was loaded into the emulsification device and droplets were formed by QX200 droplet generator. The cfDNA was amplified in a Veriti Thermal Cycler (Applied Biosystems®, Foster City, CA, USA). The amplification program is: 1 cycle of 10 minutes at 95°C, 40 cycles of 15 seconds at 94°C and 60 seconds at 60°C, 1 cycle of 10 minutes at 98°C then cooling to 12°C. Absolute quantities of cfDNA copies are determined using the [QX200™ Droplet Reader](https://www.bio-rad.com/evportal/destination/commerce/sku_detail?cmd=catProductDetail&vertical=LSR&productID=186-4003) and the QX Manager 1.2 Standard Edition software. Quantification is validated when the TPC, NEC and NAC controls are correct, the number of droplets is greater than 8000 and the ICE quantification of each well is in accordance with the expected value.

cfDNA qualification and quantification by BIABooster

Fragment analysis was performed using BIABooster technology (Adelis®, Labege, France). The technology is operated automatically on a commercial capillary electrophoresis instrument using electro-hydrodynamic actuation. All the samples were treated by RNase 0.1U/μl, before analyzing. BIABooster technology enabled analysis of cfDNA fragments between 75 and 1649 bp, according to manufacturer’s protocol. A reference ladder determines the sizes at each pass. Four peaks and seven areas (<75pB, 80-120pb, 75-111pb, 111-240pb, 240-370pb, 370-580pB, 580-1650pb, >1650pB) are identified. The cfDNA concentration (pg/µl) is measured under each area.

dd-cfDNA determination by NGS AlloSeq cfDNA®

The AlloSeq cfDNA kit (CareDx Pty Ltd, WA, Australia) enables relative quantification of the donor-derived cell-free DNA (dd-cfDNA) in a cfDNA sample derived from a transplant recipient. Following cfDNA extraction from plasma, the cfDNA is amplified using multiplex PCR that includes PCR primers for 202 single nucleotide polymorphisms. The resulting PCR products are sequenced on an MiSeq (Illumina, Inc. San Diego, CA) sequencing instrument, and the sequence data is analyzed using the CareDx AlloSeq cfDNA software. Genotyping of recipients and donors is not necessary because cfDNA present as the minor cfDNA contributor is assigned as ‘donor-derived’. More precisely, the AlloSeq cfDNA kit is for 24 samples. For one sample the reaction volume is 40µL with 4µL of reverse index primers, 4μL of direct index primers, 13µL of AlloSeq cfDNA PCR Mix, 0.8µL of AlloSeq cfDNA PCR Enzyme, 2.2µL of AlloSeq cfDNA SNP Primer Pool and 16µL of DNA at 0.625 ng/μL. The amplification protocol is unique and corresponds to 98°C for 3 minutes, 8 cycles of 96°C for 15 seconds + 70°C for 5 seconds + 57°C for 60 seconds + 72°C for 30 seconds, following by 12 cycles of 96°C for 15 seconds and 72°C for 60 seconds, 72°C for 2 minutes and 10°C. Then, a double purification step on beads is done and finally a denaturation step with 0.2N sodium hydroxide for the sequencing reaction which is performed using the MiSeq v3 reagent kit, 150 cycles. Total result delivery time is about one day with 2 hours of hands-on time and 17 hours of NGS sequencing. Data are analyzed using CareDx AlloSeq cfDNA software which automatically calculates dd-cfDNA relative quantification. In each run, a positive control (previous sample with known dd-cfDNA value) and a negative control (water) are tested.

Identification of HLA antibodies against the donor (DSA)

Identification of antibody specificity was carried out using a LABScan 200 Flow analyzer (Luminex Corporation, Austin, TX). The reagents used were LABScreen Single Antigen HLA Class I and Class II (One Lambda, Canoga Park, CA). The tests were carried out according to the manufacturers’ instructions, and the analysis was performed with HLA Fusion 4.4.0 software. The confirmation of a DSA is done by comparing the specificities of the anti-HLA antibodies with the typing of the donor done in FluoGene® SSP-PCR technique (Inno-train, Kronberg, Germany) and confirmed by NGS technology (NGmix®, EFS).
